# Long-term outcomes of venetoclax and ibrutinib in Japanese patients with relapsed/refractory mantle cell lymphoma

**DOI:** 10.1007/s10147-025-02865-4

**Published:** 2025-09-02

**Authors:** Hideki Goto, Satoshi Ito, Masahiro Kizaki, Masaki Yamaguchi, Noriko Fukuhara, Koji Kato, Toko Saito, Yasuhito Terui, Tomomi Soshin, Natsuko Satomi-Tsushita, Hideyuki Honda, Chen Qian, Koji Izutsu

**Affiliations:** 1 Hokkaido University Hospital, Kita-14, Nishi-5, Kita-ku, Sapporo-shi, Hokkaido, 060-8648 Japan; 2 Yamagata University Hospital, Yamagata, Japan; 3 Saitama Medical Center, Saitama Medical University, Saitama, Japan; 4 Ishikawa Prefectural Central Hospital, Ishikawa, Japan; 5 Tohoku University Hospital, Miyagi, Japan; 6 Kyushu University Hospital, Fukuoka, Japan; 7 Aichi Cancer Center, Aichi, Japan; 8 Saitama Medical University Hospital, Saitama, Japan; 9 AbbVie, Tokyo, GK Japan; 10 AbbVie, Inc, North Chicago, IL USA; 11 National Cancer Center Hospital, Tokyo, Japan

**Keywords:** Venetoclax, Ibrutinib, Relapsed/refractory, Mantle cell lymphoma

## Abstract

**Background:**

Patients with relapsed/refractory (R/R) mantle cell lymphoma (MCL) face a poor prognosis in the absence of effective treatment options. Ibrutinib plus venetoclax demonstrated high response rates and a tolerable safety profile in the primary analysis of the Phase 2, M20-075 study (NCT04477486) in Japanese patients with R/R MCL. We report updated efficacy and safety from this study with longer follow-up.

**Methods:**

Patients received 560 mg ibrutinib and 400 mg venetoclax (5-week ramp-up to 400 mg) once daily for up to 104 weeks followed by ibrutinib monotherapy. Primary endpoint was Independent Review Committee-assessed complete response (CR) rate. Secondary endpoints included overall response rate (ORR), duration of response (DOR), undetectable minimal residual disease (uMRD) in patients achieving CR, progression-free survival (PFS), overall survival (OS), and safety.

**Results:**

After a median follow-up of 37.2 months, 13 patients had received ibrutinib plus venetoclax, 8 (62%) remained on ibrutinib monotherapy, and 9 (69%) completed 24 months of venetoclax. ORR was 83% (10/12 [per-protocol population]; all CR); median DOR was not reached. All 6 patients positive for MRD at baseline who achieved CR had uMRD. Median PFS and OS were not reached. Most frequent Grade ≥ 3 treatment-emergent adverse events (TEAEs) were neutropenia (46%) and leukopenia (23%); one TEAE leading to treatment discontinuation was squamous cell carcinoma unrelated to treatment. There were no cases of tumor lysis syndrome or TEAEs leading to death.

**Conclusion:**

Long-term follow-up of ibrutinib plus venetoclax showed prolonged efficacy and a well-tolerated safety profile in Japanese patients with R/R MCL.

**Supplementary Information:**

The online version contains supplementary material available at 10.1007/s10147-025-02865-4.

## Introduction

Mantle cell lymphoma (MCL) is an aggressive and rare form of non-Hodgkin lymphoma that is commonly diagnosed at an advanced stage and occurs in approximately 3% of all lymphoma cases in Japan [1–3]. Despite high initial response rates to frontline treatment (combination chemotherapy with rituximab for advanced stage in Japan [1]), patients often experience relapsed/refractory (R/R) disease and require subsequent therapy. The management of R/R MCL remains challenging, and in the absence of a cure, patients face a poor prognosis with a median overall survival (OS) of 3–4 years [3, 4]. Owing to substantial progress in the field, a shift in the treatment paradigm for MCL from chemotherapy to targeted therapies offers promising treatment options for patients with R/R disease. Ibrutinib, a first-in-class oral Bruton’s tyrosine kinase inhibitor has demonstrated promising efficacy in patients with R/R MCL and was approved in Japan in 2016 in this patient population. Despite the marked advancements, new treatment strategies are needed for substantial improvement in outcomes, because the overall prognosis remains poor in patients with R/R MCL. Venetoclax is a highly selective, potent, oral B-cell lymphoma 2 (BCL-2) inhibitor [3]. High response rates and a tolerable safety profile were observed following venetoclax monotherapy in patients with R/R MCL [2, 5, 6]. Venetoclax in combination with ibrutinib has shown synergistic antitumor activity and significantly improved efficacy compared with historical controls in patients with R/R MCL [7, 8]. In the multinational, double-blind, Phase 3 SYMPATICO study (NCT03112174), patients with R/R MCL who received 1–5 prior lines of therapy were randomized to oral ibrutinib 560 mg once daily combined with oral venetoclax (with a standard 5-week ramp-up schedule from 20 mg to a target dose of 400 mg to reduce the risk of tumor lysis syndrome [TLS]) once daily or placebo for 2 years, followed by ibrutinib monotherapy [9]. After a median follow-up of 51.2 months, this regimen resulted in a significantly improved median progression-free survival (PFS) and CR rate compared with ibrutinib plus placebo [9].

M20-075 is a Phase 2 study (NCT04477486) of venetoclax with ibrutinib in Japanese patients with R/R MCL [3]. In the primary results, this combination demonstrated high response rates and a tolerable safety profile in patients who received a median of 2 prior lines of therapy [3]. More specifically, with a median follow-up of 9.6 months, independent review committee (IRC)-assessed complete response (CR) rate and overall response rate (ORR) were 83% each (per protocol set [PPS]). No dose-limiting toxicities, TLS, or deaths related to treatment-emergent adverse events (TEAEs) were observed. However, the long-term efficacy and safety of venetoclax with ibrutinib in this patient population was unclear in the primary analysis due to the short follow-up period [3].

The aim of this analysis is to report on the long-term efficacy and safety of venetoclax in combination with ibrutinib from the M20-075 study, extending beyond what has been previously published, with insights from prolonged follow-up with a median of 37.2 months (range 2.3–43.3).

## Patients and methods

### Study design and patients

M20-075 (NCT04477486) is an open-label, single-arm, Phase 2 study. Study design and patients have been previously described in detail (Fig. S1) [3]. Briefly, eligible patients were aged ≥ 20 years and had pathologically confirmed R/R MCL and at least one measurable disease site. Patients had to have received ≤ 5 prior lines of therapy, including ≥ 1 prior rituximab/anti-CD20-containing regimen. The full analysis set (FAS) comprised patients (N = 13) who received ≥ 1 dose of study drug. The PPS (n = 12) excluded FAS patients with non-evaluable disease at baseline based on assessment by IRC.

### Treatment and assessments

Patients received 560 mg oral ibrutinib and 400 mg oral venetoclax (with a 5-week dose ramp-up schedule to mitigate the risk of TLS) once daily for up to 24 months followed by ibrutinib monotherapy until disease progression, unacceptable toxicity, or withdrawal of consent. Venetoclax ramp-up entailed 4 dose increases weekly, with patients receiving a starting dose of 20 mg, followed by 50 mg, 100 mg, 200 mg, and 400 mg venetoclax. Recommended strategies for dose modifications of venetoclax due to adverse events (AEs) during treatment, including TLS, and dose modifications are listed in Tables S1 and S2 [10]. Definitions of laboratory and clinical TLS per Howard’s criteria are listed in Table S3 [11].

Efficacy was determined by the CR rate as best overall response; ORR (defined as the rate of best overall response of [CR + partial response]); duration of response (DOR, time from first recorded response to disease progression or death); undetectable minimal residual disease (uMRD) rate in patients achieving CR (investigator-assessed); PFS (time from the first dose of study drug to data about investigator-assessed disease progression per Lugano classification or death; overall survival (OS, time from the initial dose of any study treatment to death of any cause). MRD was measured by flow cytometry in bone marrow aspirate and/or peripheral blood from patients who have achieved a CR with a threshold of < 0.05% MCL cells per total white blood cells. Safety was evaluated throughout the study with grading TEAEs and severity according to the National Cancer Institute Common Terminology Criteria for Adverse Events version 5.0. All TEAEs were reported from the time of study drug administration until 30 days after discontinuation of study. Severe TEAEs were reported as Grade ≥ 3. Serious TEAEs were AEs with a substantial impact on the patient's health, potentially including life-threatening situations, hospitalization, or disability, regardless of its severity grade. Criteria for serious TEAEs are listed in Table S4.

The data cutoff for the efficacy and safety analysis reported here was July 1, 2024, representing a median follow-up duration of 37.2 months (range 2.3–43.3) and providing a long-term efficacy and safety evaluation of ibrutinib plus venetoclax in Japanese patients with R/R MCL.

### Statistical analyses

The FAS was used for all efficacy endpoints (except endpoints based on assessment by IRC), safety, PK, and baseline analysis. The PPS was used for endpoints based on assessment by IRC and also used for investigator assessment endpoints. The estimate and 95% confidence interval (CI) for the CR rate, ORR, and uMRD rate were based on exact binomial distribution. The CR rate was compared with a historical control threshold of 12.5% observed with ibrutinib monotherapy in Japanese patients with R/R MCL using the exact binomial test at a 1-sided overall significance level of 0.025 [12]. For time-to-event endpoints, survivorship function was estimated by using the Kaplan–Meier product-limit method. All analyses were performed using SAS Version 9.4 or later under a UNIX operating system.

## Results

### Patient characteristics

A total of 13 patients received ibrutinib plus venetoclax. Most patients in the FAS were male (77%) and had an Eastern Cooperative Oncology Group performance status of 0 (85%). The median age was 71 years (range 59–81). At baseline, 3 patients (23%) had bone marrow involvement and 3 (23%) had gastrointestinal involvement; 5 patients (38%) had bulky disease ≥ 5 cm and 3 (23%) had a blastoid morphology. Median number of prior lines of therapy was 2 (range 1–3) (Table 1).

**Table 1:** Patient demographics and baseline characteristics in the per protocol set and the full analysis set

Characteristic	PPS (n = 12)	FAS (N = 13)
Age		
Median (range), years	71 (59–81)	71 (59–81)
≥ 65, n (%)	10 (83)	11 (85)
Sex, n (%)		
Male	9 (75)	10 (77)
Relapsed/refractory disease, n (%)		
Relapsed	12 (100)	13 (100)
Number of prior regimens		
Median (range)	2 (1–3)	2 (1–3)
1, n (%)	1 (8)	2 (15)
2, n (%)	7 (58)	7 (54)
≥ 3, n (%)	4 (33)	4 (31)
Prior ASCT, n (%)	3 (25)	3 (23)
ECOG PS, n (%)		
0	10 (83)	11 (85)
1	2 (17)	2 (15)
Bone marrow involvement (at baseline), n (%)		
Yes	2 (17)	3 (23)
GI involvement (at baseline), n (%)		
Yes	3 (25)	3 (23)
Bulky disease, n (%)		
≥ 5 cm	4 (33)	5 (38)
MIPI score, n (%)		
Low	2 (17)	3 (23)
Intermediate	7 (58)	7 (54)
High	3 (25)	3 (23)
MCL histology, n (%)		
Typical	6 (50)	7 (54)
Blastoid	3 (25)	3 (23)
Pleomorphic	2 (17)	2 (15)
Other/unknown	2 (17)	2 (15)
TLS risk category, n (%)		
Low	7 (58)	7 (54)
High	5 (42)	6 (46)
Lugano classification, n (%)		
Stage II	2 (17)	3 (23)
Stage III	2 (17)	2 (15)
Stage IV	8 (67)	8 (62)

*ASCT* autologous stem cell transplant, *ECOG PS* Eastern Cooperative Oncology Group performance status, *FAS* full analysis set; GI, gastrointestinal, *MCL* mantle cell lymphoma, *MIPI* Mantle Cell Lymphoma International Prognostic Index, *PPS* per protocol set, *TLS* tumor lysis syndrome

### Efficacy outcomes

The median duration of treatment with ibrutinib plus venetoclax was 32.9 months (range 0.2–41.6) (venetoclax, 23.1 months [range 0.2–24.4]; ibrutinib, 32.9 months [range 0.2–41.6]). At the data cutoff, 9 of 13 patients completed 24 months of venetoclax treatment, and 4 patients discontinued venetoclax: 1 patient (Patient-04) discontinued due to squamous cell carcinoma (SCC) of the lung, 2 patients (Patients-012 and −013) discontinued due to disease progression, and 1 patient (Patient-08) discontinued due to physician decision. Patients who interrupted venetoclax after completing 24 months of treatment and did not meet the eligibility criteria to resume therapy (although those patients could resume venetoclax treatment after interruption) were reported as “completed 24 months of venetoclax”. For instance, Patient-10 was reported as such although venetoclax had been interrupted for an extended period of time due to Grade 3 stomatitis (from Day 338–512) and Grade 2 malaise (Day 449). Eight patients were still receiving ibrutinib, whereas 5 discontinued treatment: 1 patient discontinued due to SCC of the lung, 3 patients (Patients-01, 012, and 013) discontinued due to disease progression, and 1 patient discontinued due to withdrawal of consent (Patient-04).

**Fig. 1:**
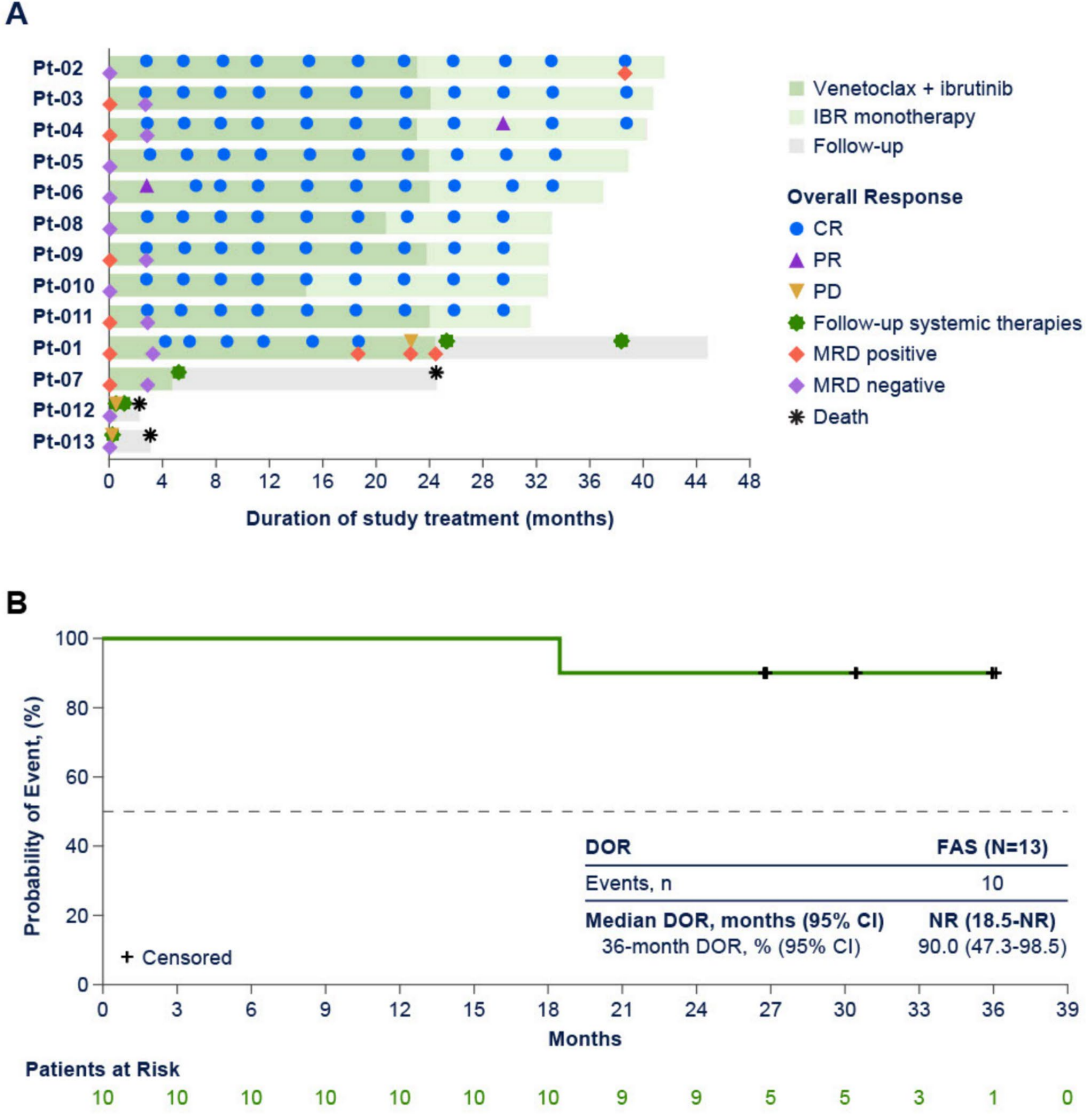
Overall response and time on study (**A**)^a^ and duration of response (**B**) by investigator (full analysis set, N = 13). *PB*, peripheral blood, *CR* complete response, *DOR* duration of response, *MRD* minimum residual disease, *NR* not reached, *PD* progressive disease, *PR* partial response, *Pt* patient, *uMRD*, undetectable minimum residual disease. ^a^MRD by PB and MRD by PB from baseline to the earliest uMRD and subsequent positive MRD were included in the swim lane plot

**Fig. 2:**
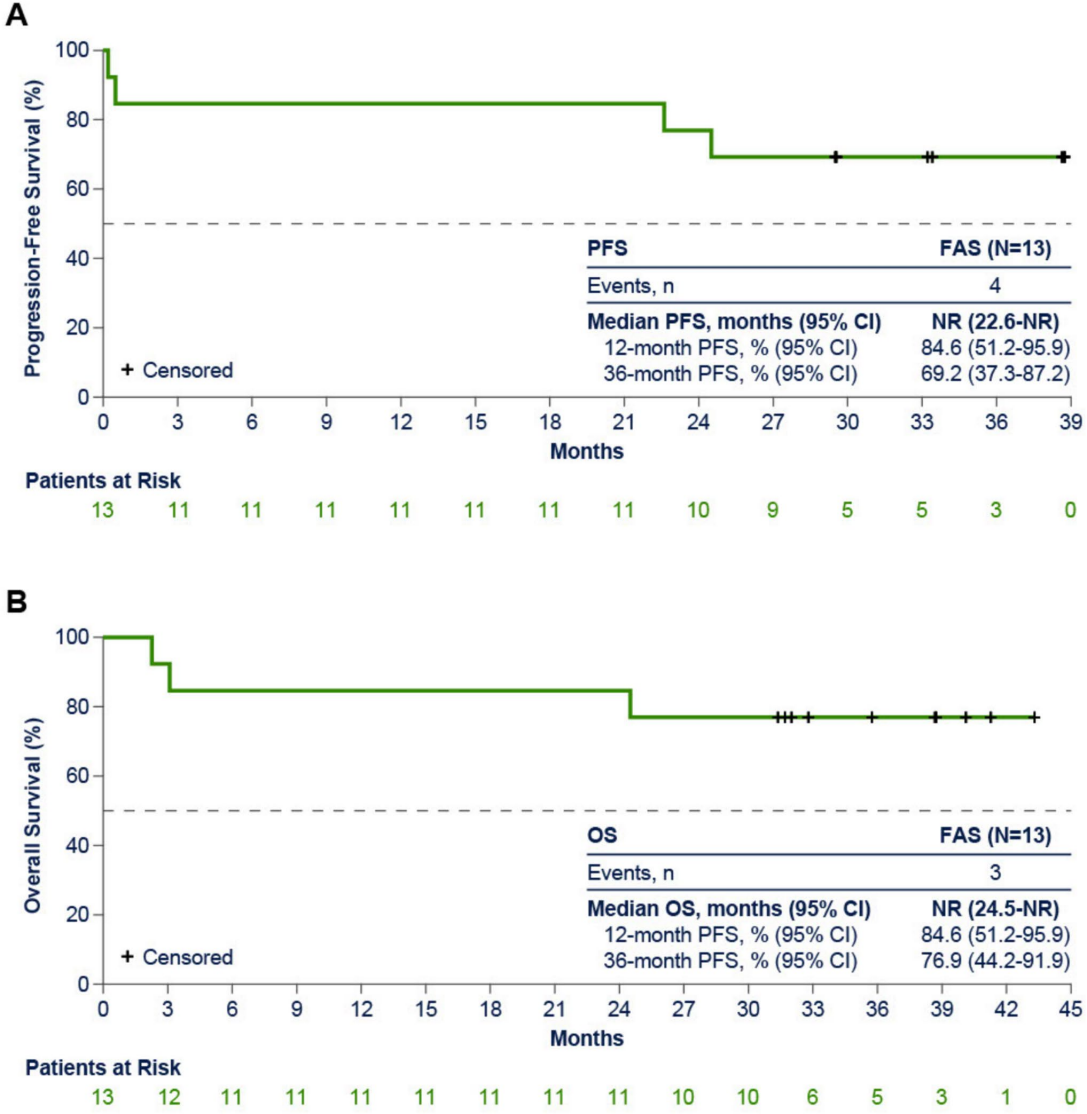
Progression-free survival (**A**) and overall survival (**B**) (full analysis set, N = 13). *FAS*, full analysis set, *NR* not reached, *OS* overall survival, *PFS* progression-free survival

After a median follow-up of 37.2 months (range 2.3–43.3), ORR was 83% (10 of 12 patients in the PPS; 95% CI [51.6–97.9]), with all patients achieving CR (Table 2); event-free rate for DOR (95% CI) was 90% (47.3–98.5) at 36 months and median DOR was not reached (Table 2). Overall response and treatment duration in the FAS is summarized in the swim lane plot: 9 of 13 (69%) completed 24 months of venetoclax treatment, and 8 of 13 (62%) remained on ibrutinib monotherapy (Fig. 1). Nine of 10 patients who responded to treatment achieved a CR by Week 13 visit, and 8 of 10 patients who achieved a CR remained in CR after completion of 24 months of venetoclax (Fig. 1). A total of 7 patients were positive for MRD in peripheral blood (PB; n = 5) and/or bone marrow (BM; n = 2) at baseline; 6 of those patients achieved CR and all had uMRD in PB and/or BM. Five of 5 patients achieved uMRD in PB by Week 13 visit and 2 of 2 patients achieved uMRD in BM when achieved CR. Median PFS (Fig. 2A; Table 2) and OS (Fig. 2B; Table 2) were not reached in the FAS population. The 12-month PFS and OS (95% CI) were 84.6% (51.2–95.9), whereas the 36-month PFS and OS were 69.2% (37.3–87.2) and 76.9% (44.2–91.9), respectively (Table 2).

**Table 2:** Summary of efficacy in the per protocol set and full analysis set

Outcome	PPS (n = 12)	FAS (N = 13)
Best overall response, n (%)		
CR	10 (83)	10 (77)
PR	0	0
SD	0	0
PD	2 (17)	2 (15)
NE	0	1 (8)
ORR, n (%) [95% CI]	10 (83.3) [51.6–97.9]	10 (77) [46.2–95.0]
Median DOR, months		
(95% CI)^a^	NR (18.5–NE)	NR (18.5–NE)
36-month DOR, %		
(95% CI)	90.0 (47.3–98.5)	90.0 (47.3–98.5)
Median PFS, months (95% CI)	NR (0.5–NE)	NR (22.6–NE)
12-month PFS, % (95% CI)	83.3 (48.2–95.6)	84.6 (51.2–95.9)
36-month, PFS, % (95% CI)	75.0 (40.8–91.2)	69.2 (37.3–87.2)
Median OS, months (95% CI)	–	NR (24.5–NE)
12-month OS, %		
(95% CI)	–	84.6 (51.2–95.9)
36-month OS,%		
% (95% CI)	–	76.9 (44.2–91.9)

*CR* complete response, *DOR* duration of response, *FAS* full analysis set, *NE* not estimable, *NR* not reached, *ORR* overall response rate, *OS* overall survival, *PD* progressive disease, *PPS* per protocol set, *PR* partial response, *SD* stable disease

^a^Assessed by investigator in patients with CR

As of the data cut-off, there were 3 patients with progressive disease (PD), in two of those cases (Patients-012 and −013) PD occurred early on during the course of treatment with venetoclax, whereas in 1 patient (Patient-01) PD occurred after nearly completing 24 months of venetoclax treatment (Fig. 1). More specifically, Patient-012, a 70-year-old male who received 3 prior lines of previous systemic therapy, had a pleomorphic MCL histology, Stage IV per Lugano classification with bulky disease [6.7 cm], and intermediate Mantle Cell Lymphoma International Prognostic Index [MIPI] score) discontinued venetoclax due to PD, 15 days after treatment initiation, and died nearly 2 months (54 days) after the last dose. Patient-013 (a 71-year-old male who received 2 lines of prior systemic therapy, had a typical MCL histology, Stage IV per Lugano classification with bulky disease [13.262 cm], and high MIPI score) also discontinued venetoclax due to PD, 6 days after treatment initiation, and died nearly 3 months (88 days) after the last dose. These 2 patients experienced rapid clinical progression leading to treatment discontinuation during the venetoclax ramp-up period. Patient-01 (a 69-year-old female who received 1 line of prior systemic therapy, had a blastoid MCL histology, Stage IV per Lugano classification without bulky disease [3.405 cm], high MIPI score, and baseline bone marrow involvement) achieved CR and MRD negativity at the initial Week 13 disease assessment. As of Day 463, venetoclax dosing was reduced to 200 mg due to Grade 3 neutropenia. Disease assessment in Week 81 showed this patient maintained CR but was confirmed positive for MRD in PB. From Day 585 to Day 610, venetoclax was interrupted due to COVID-19, and subsequently resumed at 200 mg starting on Day 611; on Day 688 this patient developed PD.

### Safety outcomes

All 13 patients experienced any-grade TEAEs. The most common TEAEs experienced by ≥ 20% patients were diarrhea and neutropenia (54% each) followed by constipation, leukopenia, and pyrexia (38% each), anemia, thrombocytopenia and skin infection (31% each), decreased appetite, hyperkalemia, nausea, muscle spasms and pneumonia (23% each) (Table 3). Grade ≥ 3 TEAEs were reported in 62% of patients (8/13), with the most frequent being neutropenia (46%) and leukopenia (23%) (Table 3). Serious TEAEs occurred in 46% of patients (6/13); most were Grade 3 (including 2 cases of COVID-19 pneumonia), with the exception of 1 case of Grade 4 sepsis and 1 case of Grade 4 neutropenia reported in the primary analysis (Table 3). TEAEs led to venetoclax interruption and dose reduction in 62% (8/13) and 54% (7 of 13) of patients, respectively. The primary cause for a venetoclax dose reduction was neutropenia (4 of 13 patients; 31%). One TEAE that led to venetoclax discontinuation was SCC of the lung deemed unrelated to the treatment and was previously reported [3]. TEAEs led to ibrutinib interruption and dose reduction in 62% (8/13) and 38% (5 of 13) of patients, respectively. Similar to venetoclax, SCC of the lung was the only TEAE that led to ibrutinib discontinuation and was not related to treatment (Table 4). Three deaths beyond the 30-day safety follow-up period were reported during the course of the study, none related to TEAEs; 2 patients died of disease progression, and 1 died of SCC of the lung. Following the primary analysis, there have been no AEs leading to study drug discontinuation and no deaths. There were no cases of TLS.

**Table 3:** Most common TEAEs (full analysis set, N = 13)

TEAEs, n (%)^a^	Any grade	Grade ≥ 3	Serious TEAEs^b^
Any	13 (100)	8 (62)	6 (46)
Hematological			
Neutropenia	7 (54)	6 (46)	1 (8) (Grade 4)
Leukopenia	5 (38)	3 (23)	0
Anemia	4 (31)	1 (8)	0
Thrombocytopenia	4 (31)	1 (8)	0
Non-hematological			
Diarrhea	7 (54)	0	0
Constipation	5 (38)	0	0
Pyrexia	5 (38)	1 (8)	1 (8) (Grade 3)
Skin infection	4 (31)	0	0
Decreased appetite	3 (23)	0	0
Hyperkalemia^c^	3 (23)	0	0
Nausea	3 (23)	0	0
Pneumonia	3 (23)	0	1 (8) (Grade 3)
Muscle spasms	3 (23)	0	0
COVID-19	2 (15)	1 (8)	1 (8) (Grade 3)
COVID-19 pneumonia	2 (15)	2 (15)	2 (15) (Grade 3)
Hypertension	2 (15)	1 (8)	0
Pneumonia bacterial	2 (15)	1 (8)	1 (8) (Grade 3)
Bile duct stenosis	1 (8)	1 (8)	1 (8) (Grade 3)
Hemorrhoids	1 (8)	1 (8)	1 (8) (Grade 3)
Femoral neck fracture	1 (8)	1 (8)	1 (8) (Grade 3)
Glaucoma	1 (8)	1 (8)	1 (8) (Grade 3)
Hypokalemia	1 (8)	1 (8)	0
Intervertebral discitis	1 (8)	1 (8)	1 (8) (Grade 3)
Lower gastrointestinal hemorrhage	1 (8)	1 (8)	1 (8) (Grade 3)
Organizing pneumonia	1 (8)	1 (8)	1 (8) (Grade 3)
Pancreatitis	1 (8)	1 (8)	1 (8) (Grade 3)
Pneumothorax	1 (8)	1 (8)	1 (8) (Grade 3)
Sepsis	1 (8)	1 (8)	1 (8) (Grade 4)
Squamous cell carcinoma of lung	1 (8)	1 (8)	1 (8) (Grade 3)
Subdural hematoma	1 (8)	1 (8)	1 (8) (Grade 3)
Upper respiratory tract infection	1 (8)	1 (8)	1 (8) (Grade 3)

*TEAE* treatment-emergent adverse event, *TLS* tumor lysis syndrome

^a^TEAEs of any grade reported in ≥ 20% of patients, or Grade ≥ 3 TEAEs reported in any patient

^b^Some patients experienced multiple serious TEAEs

^c^Not related to TLS by investigators and did not meet Howard’s criteria[11]

**Table 4:** Ibrutinib and venetoclax dose modifications due to TEAEs (full analysis set, N = 13)

TEAEs, n (%)	FAS (N = 13)	
	Ibrutinib	Venetoclax
TEAE leading to discontinuation, n (%)	1 (8)	1 (8)
Squamous cell carcinoma of the lung	1 (8)	1 (8)
TEAE leading to dose reduction, n (%)	5 (38)	7 (54)
Atrial fibrillation	1 (8)	0
Leukopenia	1 (8)	1 (8)
Lower gastrointestinal hemorrhage	1 (8)	0
Pneumonia	1 (8)	0
Rash pruritic	1 (8)	1 (8)
Sepsis	1 (8)	1 (8)
Subdural hematoma	1 (8)	0
COVID-19 pneumonia	0	1 (8)
Nausea	0	1 (8)
Neutropenia	0	4 (31)
TEAE leading to interruption, n (%)	8 (62)	8 (62)
COVID-19	2 (15)	2 (15)
Pneumonia bacterial	2 (15)	1 (8)
Pyrexia	2 (15)	1 (8)
ALT increased	1 (8)	1 (8)
Bile duct stenosis	1 (8)	1 (8)
Cholangitis	1 (8)	0
Cholangitis acute	1 (8)	1 (8)
COVID-19 pneumonia	1 (8)	1 (8)
C-reactive protein increased	1 (8)	1 (8)
Diarrhea	1 (8)	1 (8)
Epistaxis	1 (8)	0
Erythema multiform	1 (8)	1 (8)
Femoral neck fracture	1 (8)	0
Gastrointestinal hemorrhage	1 (8)	1 (8)
Hematuria	1 (8)	0
Hemorrhoids	1 (8)	1 (8)
Hyperkalemia	1 (8)	1 (8)
Hypersensitivity	1 (8)	1 (8)
Intervertebral discitis	1 (8)	1 (8)
Leukopenia	1 (8)	1 (8)
Lower gastrointestinal hemorrhage	1 (8)	1 (8)
Neutropenia	1 (8)	2 (15)
Organizing pneumonia	1 (8)	0
Pancreatitis	1 (8)	1 (8)
Pneumonia	1 (8)	0
Pneumothorax	1 (8)	0
Rash pruritic	1 (8)	1 (8)
Sepsis	1 (8)	1 (8)
Subdural hematoma	1 (8)	1 (8)
Upper respiratory tract infection	1 (8)	0
White blood cell count decreased	1 (8)	1 (8)
Malaise	0	1 (8)
Squamous cell carcinoma of lung	0	1 (8)
Stomatitis	0	1 (8)

*ALT* alanine transferase, *FAS* full analysis set, *TEAE* treatment-emergent adverse event

The most common TEAEs of any grade in the first year (N = 13) were diarrhea (54%), neutropenia and leukopenia (38% each); after 1–2 years (n = 10), neutropenia (50%) and leukopenia and anemia (40% each) occurred most frequently, whereas anemia (40%), thrombocytopenia, diarrhea, constipation, and decreased appetite (30% each) were most commonly reported after ≥ 2 years (n = 10). The Grade ≥ 3 TEAE most commonly experienced in the first year was neutropenia (31%). After 1–2 years, the most common Grade ≥ 3 TEAEs were neutropenia (40%) and leukopenia (30%), whereas decreased appetite (20%) was most commonly reported after ≥ 2 years (Table 5).

**Table 5:** Prevalence of most common TEAEs over time (full analysis set, N = 13)

TEAEs, n (%)^a^		0–1 year (N = 13)			1–2 years (n = 10)			> 2 years (n = 10)	
	Any grade	Grade ≥ 3	Serious TEAEs	Any grade	Grade ≥ 3	Serious TEAEs	Any grade	Grade ≥ 3	Serious TEAEs
Hematological									
Neutropenia	5 (38)	4 (31)	1 (8)	5 (50)	4 (40)	0	1 (10)	1 (10)	0
Leukopenia	5 (38)	1 (8)	0	4 (40)	3 (30)	0	2 (20)	1 (10)	0
Anemia	1 (8)	0	0	4 (40)	1 (10)	0	4 (40)	1 (10)	0
Thrombocytopenia	3 (23)	0	0	3 (30)	1 (10)	0	3 (30)	1 (10)	0
Non-hematological									
Diarrhea	7 (54)	0	0	2 (20)	0	0	3 (30)	0	0
Constipation	3 (23)	0	0	3 (30)	0	0	3 (30)	0	0
Nausea	3 (23)	0	0	2 (20)	0	0	1 (10)	0	0
Pyrexia	3 (23)	0	0	0	0	0	2 (20)	1 (10)	1 (10)
Malaise	1 (8)	0	0	2 (20)	0	0	2 (20)	0	0
Skin infection	3 (23)	0	0	2 (20)	0	0	2 (20)	0	0
Pneumonia	2 (15)	0	0	1 (10)	0	0	2 (20)	1 (10)	1 (10)
Impetigo	2 (15)	0	0	2 (20)	0	0	1 (10)	0	0
Fall	0	0	0	2 (20)	1 (10)	0	0	0	0
Decreased appetite	1 (8)	0	0	2 (20)	1 (10)	0	3 (30)	2 (20)	0
Hyperkalemia	3 (23)	0	0	0	0	0	0	0	0
Muscle spasms	3 (23)	0	0	2 (20)	0	0	1 (10)	0	0
Hypertension	2 (15)	1 (8)	0	2 (20)	1 (10)	0	1 (10)	1 (10)	0
Atrial fibrillation	2 (15)	0	0	0	0	0	0	0	0
Stomatitis	2 (15)	1 (8)	0	1 (10)	1 (10)	0	0	0	0
ALT increased	2 (15)	0	0	1 (10)	1 (10)	0	0	0	0
Arthralgia	2 (15)	0	0	1 (10)	0	0	1 (10)	0	0
Myalgia	2 (15)	0	0	0	0	0	0	0	0
Headache	2 (15)	0	0	1 (10)	0	0	1 (10)	0	0
Dry skin	2 (15)	0	0	1 (10)	0	0	1 (10)	0	0
COVID-19	0	0	0	2 (20)	1 (10)	1 (10)	0	0	0

*ALT*, alanine aminotransferase; *TEAE*, treatment-emergent adverse event

^a^TEAEs reported in ≥ 15% of patients at any time point

## Discussion

With prolonged follow-up with a median of 37.2 months (range 2.3–43.3), venetoclax in combination with ibrutinib was confirmed to be an effective long-term treatment in Japanese patients with R/R MCL. The continued high CR rate observed with longer follow-up demonstrated durable clinical benefit in this patient population treated with ibrutinib plus venetoclax [3]. The CR rate compared favorably to the AIM study (CR rate, 71%; median follow-up 16 months) and SYMPATICO study (CR rate, 54%; median follow-up, 51.2 months) [8, 9]. Furthermore, 9 of 13 patients completed 24 months of venetoclax treatment, and 8 of 13 patients remained on ibrutinib monotherapy. All 6 evaluable patients who achieved CR had uMRD in PB and/or BM. Nine of 10 patients who responded to treatment achieved a CR by Week 13, and 8 of 10 patients who achieved a CR remained in CR after completion of the 24-month treatment with venetoclax. Median DOR, PFS, and OS were not reached.

There were 3 out of 13 patients with PD during the treatment of ibrutinib and venetoclax. Although there was no clear common background characteristics among these 3 patients, the dose intensity of venetoclax was low in all 3 patients. Two of these 3 patients experienced PD prior to reaching the target dose of venetoclax during the ramp-up period. The other patient remained on long-term treatment with ibrutinib and venetoclax with the venetoclax dose reduced to 200 mg due to AE; the patient experienced PD after about 24 days of venetocax interruption due to COVID-19.

Longer exposure to venetoclax with this combination regimen demonstrated a predictable and well-tolerated safety profile. When venetoclax treatment had to be interrupted for a long period of time, the TLS risk was individually evaluated, and treatment was restarted at a reduced dose following assessment. As a result, no TLS events were observed during the long-term follow-up. There were no additional reports of treatment discontinuation due to serious TEAEs; the case of SCC (unrelated to treatment) was observed previously [3]. Analysis of prevalence of AEs over time also showed no trend toward increased toxicity with longer treatment duration and additionally indicated that hematological toxicity and GI toxicity including diarrhea and constipation were the AEs that were continuously and frequently observed in the long term treatment period (1–2 years) of ibrutinib plus venetoclax. Those AEs are consistent with the known safety profile of ibrutinib reported in the 3-year follow-up (38.7 months) of the Phase 3, international, randomized, open-label RAY study of ibrutinib monotherapy versus temsirolimus and the final results (median follow-up of 22.5 months) of a Phase 2 study of single-agent ibrutinib in patients with R/R MCL [13, 14]. The safety profile of the ibrutinib plus venetoclax regimen is also in alignment with the AEs observed in a Phase 1, first-in-human study of venetoclax monotherapy after a 38.5-month follow-up and the double-blind, Phase 3 SYMPATICO study [9, 15]. Delayed any-grade TEAEs resulting from longer treatment with ibrutinib plus venetoclax did not represent a safety concern and included constipation, pyrexia, and anemia reported in 38%, 38%, and 31% of patients, respectively. Ibrutinib has been linked to potential cardiotoxicity including atrial fibrillation and hypertension, especially following long-term use [16, 17]. In the M20-075 study, 2 cases of cardiotoxicity and 2 cases of hypertension were reported. Two patients experienced atrial fibrillation and 1 had cardiac failure. These AEs were mild in severity (Grades 1–2) and did not require any venetoclax dose modification; however, ibrutinib treatment had to be interrupted in one patient. Importantly, these events occurred early in the course of the study in 2 patients who had early clinical progression and/or SCC. Importantly, it is known that the risk of cardiotoxicity did appear to increase with the duration of treatment with ibrutinib. In addition, hypertension was reported in 2 patients and in one of the cases, it was Grade ≥ 3. None of those patients required dose modification of venetoclax and/or ibrutinib, and this AE was considered manageable.

To date, there is limited availability of data about long-term outcomes in patients with R/R MCL. In a non-Japanese Phase 2 study, the combination of venetoclax and ibrutinib has demonstrated durable responses and treatment-free remissions with an estimated 7-year PFS of 30% and OS of 43% and acceptable toxicity profile [18]. Further, the primary analysis from the Phase 3 SYMPATICO study (NCT03112174) demonstrated a significant improvement in PFS and CR rates with a favorable safety profile following venetoclax and ibrutinib treatment in patients with R/R MCL [9]. Considering these results along with the long-term outcomes of M20-075 in Japanese patients, the combination of venetoclax and ibrutinib emerges as a promising treatment option for R/R MCL in Japan.

Limitations of this study include a small sample size and the single-arm design with no control group for comparison of long-term outcomes in Japanese patients with MCL. In addition, biomarker testing including *TP53* and *BTK* mutational status was not performed. Nonetheless, findings from this analysis strengthen the evidence supporting the continued evaluation of the ibrutinib plus venetoclax combination for patients with R/R MCL, where there remains the need for novel effective treatment strategies to improve long-term outcomes.

## Supplementary Information

Below is the link to the electronic supplementary material.

Supplementary file1 (DOCX 188 kb)

## Data Availability

AbbVie is committed to responsible data sharing regarding the clinical trials we sponsor. This includes access to anonymized, individual, and trial-level data (analysis data sets), as well as other information (eg, protocols, clinical study reports, or analysis plans), as long as the trials are not part of an ongoing or planned regulatory submission. This includes requests for clinical trial data for unlicensed products and indications. These clinical trial data can be requested by any qualified researchers who engage in rigorous, independent, scientific research, and will be provided following review and approval of a research proposal, Statistical Analysis Plan (SAP), and execution of a Data Sharing Agreement (DSA). Data requests can be submitted at any time after approval in the US and Europe and after acceptance of this manuscript for publication. The data will be accessible for 12 months, with possible extensions considered. For more information on the process or to submit a request, visit the following link: https://vivli.org/ourmember/abbvie/ then select “Home”.
