# Supplementary material for: Long-term outcomes of venetoclax and ibrutinib in Japanese patients with relapsed/refractory mantle cell lymphoma
Source: Int J Clin Oncol. 2025 Sep 2;30(11):2352–61. doi: 10.1007/s10147-025-02865-4 (PMC12568793; doi:10.1007/s10147-025-02865-4)
Supplement: Supplementary file 1 — Supplementary file1 (DOCX 188 kb) [file 10147_2025_2865_MOESM1_ESM.docx]

**Long-term outcomes of venetoclax and ibrutinib in Japanese patients with relapsed/refractory mantle cell lymphoma**

**Authors**: Hideki Goto^1^, Satoshi Ito^2^, Masahiro Kizaki^3^, Masaki Yamaguchi^4^, Noriko Fukuhara^5^, Koji Kato^6^, Toko Saito^7^, Yasuhito Terui^8^, Tomomi Soshin^9^, Natsuko Tsushita Satomi^9^, Hideyuki Honda^9^, Chen Qian^10^, Koji Izutsu^11^

**Affiliations**: ^1^Hokkaido University Hospital, Hokkaido, Japan; ^2^Yamagata University Hospital, Yamagata, Japan; ^3^Saitama Medical Center, Saitama Medical University, Saitama, Japan; ^4^Ishikawa Prefectural Central Hospital, Ishikawa, Japan; ^5^Tohoku University Hospital, Miyagi, Japan; ^6^Kyushu University Hospital, Fukuoka, Japan; ^7^Aichi Cancer Center Hospital, Aichi, Japan; ^8^Saitama Medical University Hospital, Saitama, Japan; ^9^AbbVie, GK, Tokyo, Japan; ^10^AbbVie, Inc, North Chicago, IL, USA; ^11^National Cancer Center Hospital, Tokyo, Japan.

**SUPPLEMENTARY MATERIALS**

**FIGURES**

**FIGURE LEGEND**

**Figure S1.** Study design

*BOR* best overall response, *CR* complete response, *DOR* duration of response, *IRC* Independent Review Committee, *MCL* mantle cell lymphoma, *ORR* objective response rate, *OS* overall survival, *PFS* progression-free survival, *PK* pharmacokinetic, *PO* oral, *QD* once a week, *TLS* tumor lysis syndrome, *R/R* relapsed/refractory, *uMRD* undetectable minimal residual disease
^a^20 mg followed by 50 mg, 100 mg, 200 mg, and 400 mg

**TABLES**

**Table S1** Recommended dose modifications for venetoclax-related toxicities [9]

| Event | Occurrence | Action |
| --- | --- | --- |
| Tumor lysis syndrome  Blood chemistry changes or symptoms suggestive of TLS | Any | Withhold the next day's dose. If resolved within 24 to 48 h of last dose, once the toxicity has resolved to Grade 1 or baseline level, resume at the same dose |
|  |  | For any blood chemistry changes requiring more than 48 h to resolve, once the toxicity has resolved to Grade 1 or baseline level, resume at a reduced dose (Supplementary Table 2) |
|  |  | For any events of clinical TLS, once the toxicity has resolved to Grade 1 or baseline level, resume at a reduced dose following resolution (Supplementary Table 2) |
| Non-hematologic toxicities  Grade 3 or 4 | First | Interrupt venetoclax. Once the toxicity has resolved to Grade 1 or baseline level, venetoclax therapy may be resumed at the same dose. No dose modification is required |
|  | Second | Interrupt venetoclax. Once the toxicity has resolved to Grade 1 or baseline  level, resume at a reduced dose following resolution (Supplementary Table 2).  A larger dose reduction may occur at  the discretion of the investigator |
| Hematologic toxicities  Grade 3 or 4 neutropenia with infection or fever; or Grade 4 hematologic toxicities (except lymphopenia) | First | Interrupt venetoclax. To reduce the infection risks associated with neutropenia, G-CSF may be administered with venetoclax if clinically indicated. Once the toxicity has resolved to Grade 1 or baseline level, venetoclax  may be resumed at the same dose |
|  | Second and subsequent | Interrupt venetoclax. Consider using G-CSF as clinically indicated. Once the toxicity has resolved to Grade 1 or baseline level, resume at a reduced dose following resolution  (Supplementary Table 2). A larger dose reduction may occur at the discretion of the investigator. |

*G-CSF* granulocyte colony-stimulating factor, *TLS* tumor lysis syndrome

**Table S2** Dose modifications for adverse events during treatment with venetoclax [9]

| Dose at interruption | Restart dose^a^ |
| --- | --- |
| 400 mg | 300 mg |
| 300 mg | 200 mg |
| 200 mg | 100 mg |

^a^Continue the reduced dose for at least 1 week before increasing the dose
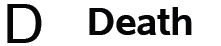


**Table S3** Definitions of laboratory and clinical TLS per Howard’s criteria^a^ [10]

| **Metabolic abnormality** | **Criteria for classification of laboratory TLS** | **Criteria for classification of clinical TLS** |
| --- | --- | --- |
| Hyperuricemia | Uric acid >8.0 mg/dl (475.8  mol/L) in adults or above the upper limit of the normal range for age in  children | - |
| Hyperphosphatemia | Phosphorus >4.5 mg/dl  (1.5 mmol/L) in adults or  >6.5 mg/dl (2.1 mmol/L) in children | - |
| Hyperkalemia | Potassium >6.0 mmol/L | Cardiac dysrhythmia or sudden death probably or  definitely caused by hyperkalemia |
| Hypocalcemia | Corrected calcium <7.0 mg/dl (1.75 mmol/L) or ionized calcium <1.12 (0.3 mmol/L)^b^ | Cardiac dysrhythmia, sudden death, seizure, neuromuscular irritability (tetany, paresthesias, muscle  twitching, carpopedal spasm, Trousseau’s sign,  Chvostek’s sign, laryngospasm, or bronchospasm), hypotension, or heart failure probably or definitely caused  by hypocalcemia |
| Acute kidney injury^c^ | N/A | Increase in the serum creatinine level of 0.3 mg/dl (26.5 µmol/L) (or a single value >1.5 times the upper limit of the age-appropriate normal range if no baseline creatinine measurement is available) or the presence of oliguria, defined as an average urine output of  <0.5 ml/kg/hour for 6 hours |

*N/A* not applicable; *TLS* tumor lysis syndrome

^a^In laboratory TLS, 2 or more metabolic abnormalities must be present during the same 24-hour period within 3 days before the start of therapy or up to 7 days afterward. Clinical TLS requires the presence of laboratory TLS plus an increased creatinine level, seizures, cardiac dysrhythmia, or death

^b^The corrected calcium level in milligrams per deciliter = measured calcium level in milligrams per deciliter + 0.8 × (4 - albumin in grams per deciliter)

^c^Acute kidney injury is defined as an increase in the creatinine level of at least 0.3 mg per deciliter (26.5 mol/L) or a period of oliguria lasting 6 hours or more. By definition, if acute kidney injury is present, the patient has clinical tumor lysis syndrome

**Table S4** Definitions of serious TEAEs

| Death of patient | An event that results in the death of a patient |
| --- | --- |
| Life-threatening | An event that, in the opinion of the investigator, would have resulted in immediate fatality if medical intervention had not been  taken. This does not include an event that would have been fatal if it  had occurred in a more severe form |
| Hospitalization or  prolongation of  hospitalization | An event that results in an admission to the hospital for any length  of time or prolongs the patient's hospital stay. This does not include  an emergency room visit or admission to an outpatient facility. |
| Congenital anomaly | An anomaly detected at or after birth, or any anomaly that results in  fetal loss |
| Persistent or significant  disability/incapacity | An event that results in a condition that substantially interferes with  the activities of daily living of a study patient. Disability is not  intended to include experiences of relatively minor medical  significance such as headache, nausea, vomiting, diarrhea, influenza,  and accidental trauma (e.g., sprained ankle). |
| Important medical event  requiring medical or surgical  intervention to prevent  serious outcome | An important medical event that may not be immediately lifethreatening or result in death or hospitalization, but based on  medical judgment may jeopardize the subject and may require  medical or surgical intervention to prevent any of the outcomes  listed above (i.e., death of patient, life-threatening, hospitalization,  prolongation of hospitalization, congenital anomaly, or persistent or  significant disability/incapacity). Additionally, any elective or  spontaneous abortion or stillbirth is considered an important  medical event. Examples of such events include allergic  bronchospasm requiring intensive treatment in an emergency room  or at home, blood dyscrasias or convulsions that do not result in  inpatient hospitalization, or the development of drug dependency or  drug abuse. |

*TEAE*, treatment-emergent adverse event

**FIGURES**

**Figure S1** Study design

*
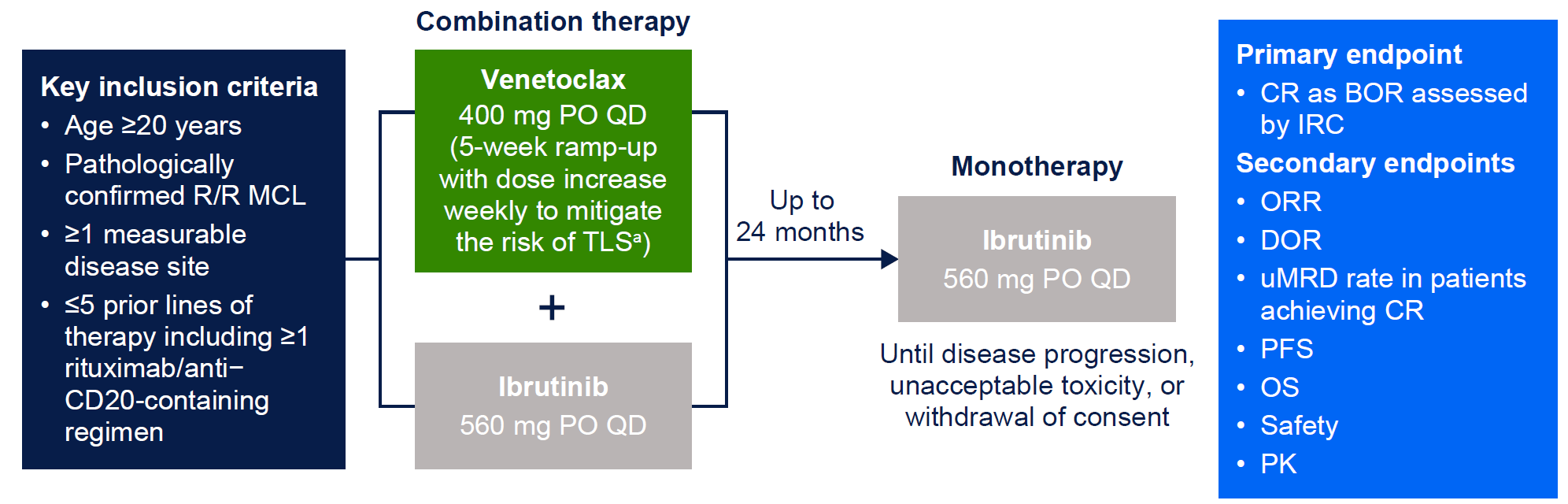
*
